# Relationship between Immediate Post-Operative Appearance and 6-Week Operative Outcome in Trichiasis Surgery

**DOI:** 10.1371/journal.pntd.0001718

**Published:** 2012-07-10

**Authors:** Shannath L. Merbs, Jennifer C. Harding, Sandra D. Cassard, Beatriz E. Munoz, Sheila K. West, Emily W. Gower

**Affiliations:** 1 Department of Ophthalmology, Wilmer Eye Institute, Johns Hopkins University School of Medicine, Baltimore, Maryland, United States of America; 2 Dana Center for Preventive Ophthalmology, Johns Hopkins University School of Medicine, Baltimore, Maryland, United States of America; 3 Department of Epidemiology and Prevention, Wake Forest School of Medicine, Winston-Salem, North Carolina, United States of America; University of Cambridge, United Kingdom

## Abstract

**Background:**

Surgical technique, including suture placement and tension, is believed to contribute to the outcome of bilamellar tarsal rotation surgery for trachomatous trichiasis. However, the immediate post-operative appearance that minimizes the chance of recurrence and other adverse outcomes has not been investigated.

**Methodology/Principal Findings:**

To explore whether the degree of correction immediately after surgery is predictive of surgical outcome at the 6-week post-operative visit, photographs taken immediately after surgery were used to predict surgical outcomes, including the severity of eyelid contour abnormality and trichiasis recurrence. Both eyelid contour abnormalities and recurrence were accurately predicted from the immediate post-operative photographs by an experienced oculoplastic surgeon 85% and 70% of the time, respectively. Participants with a “slight over-correction” that resulted in no eyelid contour abnormality and no recurrence were used to identify immediate post-operative contours that lead to a successful surgical outcome.

**Conclusions/Significance:**

The immediate post-operative eyelid contour is an important indicator of post-operative success of BLTR surgery. Based upon our findings, we developed a Surgery Photocard. This card illustrates some examples of immediate post-surgical appearances, which led to a successful outcome, as well as sub-optimal appearances, which led to poor surgical outcomes. The card also provides suggestions for improving the appearance by adjusting the suture placement or tension based upon standard oculoplastic principles.

## Author Summary

Trichiasis is a potentially blinding consequence of trachoma. The World Health Organization has promoted the bilamellar tarsal rotation (BLTR) procedure as a treatment for trichiasis from trachoma. Even if trachoma were to be eradicated today, a great number of individuals would still develop trichiasis and lose vision unless they received surgical treatment. Unfortunately, the recurrence rate after BLTR can be quite high, and surgical factors are thought to contribute to unfavorable outcomes. In this study we evaluated the relationship between immediate post-operative contour and 6-week outcomes utilizing immediate post-operative photos of 200 trichiasis surgeries. We found good agreement between the predicted and actual outcome. The analysis allowed the identification of immediate post-operative eyelid contours that were most likely to lead to successful surgery at 6 weeks, as well as contours that resulted in adverse outcomes. The description of the target degree of rotation and how to adjust the rotating sutures to best achieve this ideal should help surgeons worldwide improve their surgical technique and outcomes.

## Introduction

Despite a World Health Organization (WHO) initiative in 1998 to eliminate trachoma by 2020, this disease continues to plague poor regions of many developing countries. Even if active trachoma were to be eradicated today, millions of individuals would be at risk of blindness due to the trichiasis and corneal scarring caused by previous repeated ocular infection with *Chlamydia trachomatis.* [1] For those individuals who have already developed conjunctival scarring and trichiasis, the principle option to prevent unnecessary blindness is surgery. The WHO has endorsed the Bilamelar Tarsal Rotation (BLTR) procedure for the correction of trachomatous trichaisis (TT), [2], [3] and in many countries, this surgery is performed by non-physicians, hereafter referred to as surgical technicians. Recurrence of trichiasis after surgery is a key concern. Several studies have suggested that in addition to concurrent infection and inflammation, [4] surgical technique may contribute significantly to trichiasis recurrence. [4]–[7] Additionally, other adverse outcomes, such as eyelid contour abnormalities (ECAs) and eyelid closure defects, can occur after BLTR. The exact surgical factors that contribute to recurrence and other adverse outcomes have not been fully elucidated.

**Figure 1:**
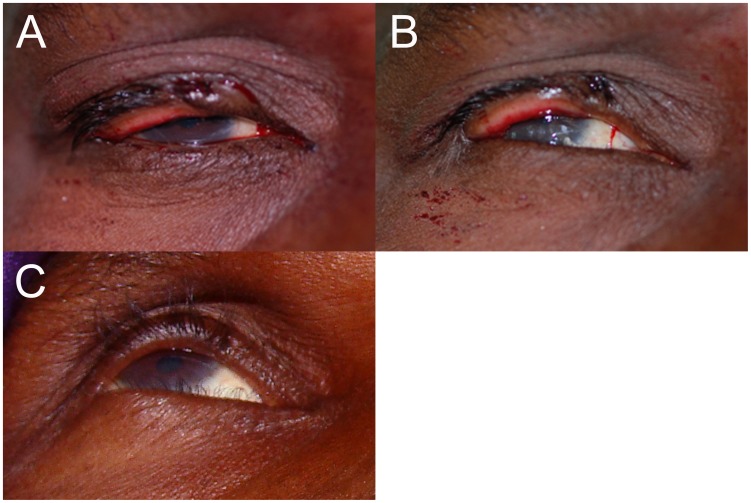
Replacement of sutures corrects nasal under-rotation. A) An immediate post-operative photograph of a participant with under-rotation of the nasal aspect of the upper eyelid margin fragment; B) the same eyelid after replacement of the rotating sutures; C) the same eyelid at the 6-week visit without recurrence (the lashes seen nasally are from the lower eyelid).

**Figure 2:**
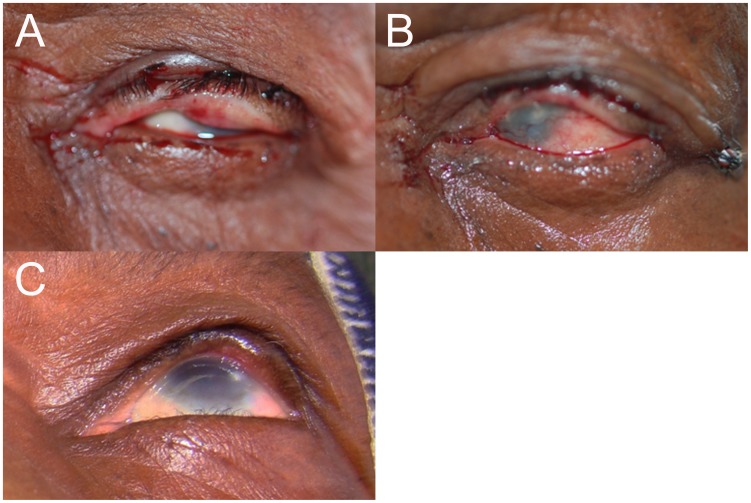
Replacement of sutures corrects eyelid margin over-rotation. A) An immediate post-operative photograph of a participant with over-rotation of the eyelid margin fragment; B) The same eyelid after replacement of the rotating sutures; C) the same eyelid at the 6-week visit with a mild ECA.

The BLTR procedure can be divided into 2 parts: the full-thickness incision creating a distal eyelid fragment containing the eyelid margin and lash line and the external rotation of this distal eyelid fragment by suture placement. The TT clamp, currently under investigation in a prospective, randomized trial, was designed to modify how the incision is created and the subsequent effect on BLTR outcome. [8] However, the importance of suture placement and tension on the BLTR outcome has yet to be investigated. The current study aims to investigate how the immediate post-operative eyelid contour relates the BLTR outcome at 6 weeks. During pre-study training to test the TT clamp against standard BLTR instrumentation, and for several weeks during the prospective, randomized clinical trial, surgical technicians performing BLTR were observed placing the rotating sutures. We noted that even when the incision was made correctly, the placement and tying of the sutures had a tremendous impact on the immediate post-operative eyelid contour and correction of the trichiasis. We, therefore, worked with the surgical technicians during the training to achieve the immediate post-operative “slight over-correction,” recommended in the WHO surgical manual. [3] When we observed significant over- or under-correction, we asked the surgical technicians to remove and replace the sutures. Typically, this correction resulted in a better eyelid contour and degree of eversion (Figures 1 and 2). Given these observations, we investigated whether the degree of correction immediately after BLTR was predictive of surgical outcome at the 6-week post-operative visit.

## Methods

### Description of participants

The ongoing Partnership for the Rapid Elimination of Trachoma (PRET) Surgery trial is designed to compare the TT clamp against the standard BLTR instrumentation. In rural southern Tanzania, 1917 participants with trichiasis and no previous trichiasis surgery in at least one eye were randomized to BLTR with either the TT clamp or standard instrumentation. A total of 3345 eyes underwent surgery as part of the study. For each participant, photographs were taken directly before and immediately after surgery, as well as at the 2- and 6-week follow up visits. At the 6-week post-operative visit, participants were examined for trichiasis recurrence, pyogenic granuloma formation and ECAs. Using a standardized grading system, photographs of each participant were graded for ECAs defined as none, mild, moderate, or severe. [9] Table 1 summarizes the grading system. Photographs were taken with the participant looking up, in order to completely visualize the eyelid margin and contour. When recurrence was present, the field grader recorded the number of lashes touching the globe as well as the location of trichiatic lashes. The location was defined by dividing the eyelid into three equal segments and describing the location of each lash base as nasal, central, or temporal. The current analysis utilizes immediate post-operative photographs and 6-week outcomes data from this ongoing study.

**Table 1:** Eyelid contour abnormality definitions.

Abnormality	Definition
**Mild**	Vertical deviation from the natural contour <1 mm in height (less than half the pupil height in daylight) and affecting <1/3 of horizontal eyelid length
**Moderate**	Vertical deviation from the natural contour 1–2 mm in height (about the pupil height in daylight) or affecting 1/3–2/3 of horizontal eyelid length
**Severe**	Vertical deviation from the natural contour >2 mm in height (more than the pupil height in daylight) or a defect >2/3 of the horizontal eyelid length

### Selection of immediate post-operative photographs

At the 6-week PRET study visit, 3341 eyelids were evaluated for postoperative outcomes. Using the PRET-study outcome data for recurrence and ECAs, 200 eyelids were randomly selected from those that were normal or had only one outcome. An equal distribution of normal, recurrence, or ECA (moderate or severe) was chosen. Therefore, the 6-week photograph set consisted of 50 normal eyelids, 50 with recurrence, 50 with moderate ECA, and 50 with severe ECA. In order for an eyelid to be selected, both immediate post-operative and 6-week follow up pictures of gradable quality were required. 16 of 3258 (0.5%) eyelids with gradable photographs were excluded because both recurrence and ECA were noted at 6 weeks. Approximately 4% of eyelids were excluded due to ungradable photographs (blurry quality, patient not looking up). Review of the pre-operative photos confirmed that in no case was an ECA present prior to BLTR surgery. The immediate post-operative photographs were evaluated in a masked fashion by a fellowship-trained oculoplastic surgeon (SLM), who was unaware of the distribution of outcomes. The evaluator was asked to record a predicted single 6-week outcome based on the appearance of the eyelid immediately after surgery - normal, recurrence (with location noted as nasal, central, and/or temporal), or ECA (moderate or severe). The primary aspect evaluated was the degree of rotation of the eyelid. When apparent under-rotation of the eyelid margin was observed in the photograph, recurrence was predicted. Conversely, when over-rotation of the eyelid margin was perceived, an ECA was predicted.

### Ethics

This study was approved by the Johns Hopkins Medicine IRB and the National Institute of Medical Research, Tanzania. Each participant provided written informed consent for participation. The study conforms to the Tenets of Helsinki.

### Statistical analysis

Eyelids with true 6-week recurrence (PRET grade) were excluded from the ECA calculations; similarly, those with ECA at 6 weeks were excluded in the recurrence calculations. The evaluator was instructed to select only one adverse outcome for each eye (either recurrence or ECA). Consequentially, if she scored an eyelid as having recurrence, automatically the eye was classified as “no abnormality” for ECA. Similarly, eyelids graded as having ECA by the evaluator automatically were classified as no recurrence. Evaluator predictions were compared with the true outcome at 6 weeks; sensitivity and specificity of the predicted outcome and corresponding 95% confidence intervals are reported.

## Results

### Recurrence of trichiasis

**Figure 3:**
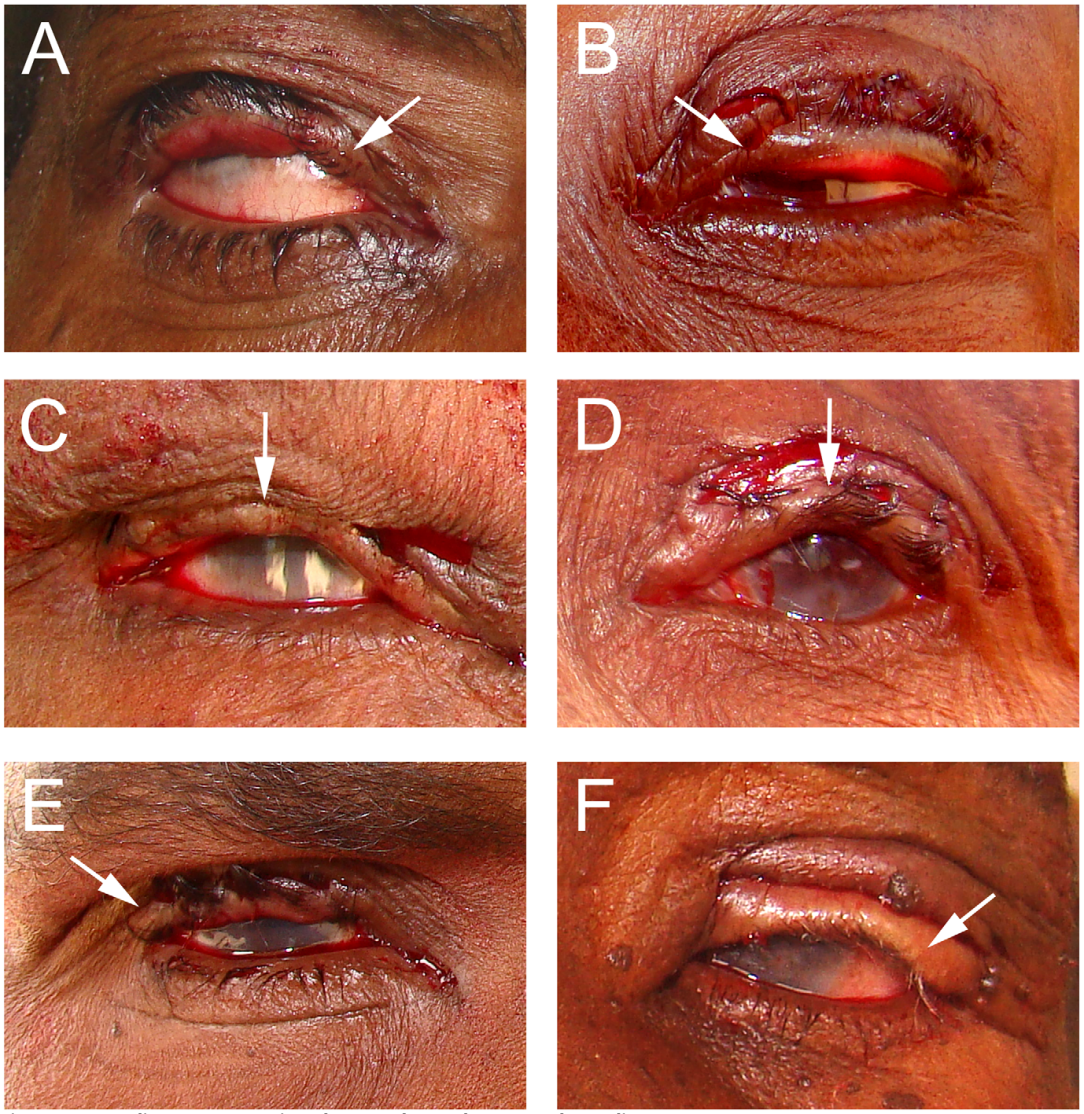
Immediate post-operative photographs used to correctly predict recurrence. Immediate post-operative photographs in 6 participants used to correctly predict A–B) nasal, C–D) central, and E–F) temporal recurrence at 6-weeks post-operatively. Arrows show area of under-rotation and subsequent recurrence.

The evaluator accurately identified 35 of 50 eyelids with recurrence (Figure 3, Table 2), with a sensitivity of 70% (95% confidence interval (CI): 57%–83%) and a specificity of 90% (95% CI: 82%–98%). Among the eyelids with recurrence, 31 had nasal recurrence, 19 had central, and 14 had temporal recurrence (some eyelids had recurrence in more than one location). The comparison of predicted and actual location(s) for recurrence is shown in Table 3. Specificity was higher than sensitivity for predicting recurrence at each location, and ranged from 78% for nasal, 90% for central, and 94% for temporal. Sensitivity for predicting the location was higher for nasal and/or central recurrence (58% each) than it was for temporal recurrence (43%) (Table 3).

**Table 2:** Comparison of predicted versus true recurrence at 6 weeks post-operatively.

Prediction*	Presence of Recurrence at 6 Weeks	
	No	Yes
**No**	45	15
**Yes**	5	35
**Sensitivity (95% CI)**	70% (57%–83%)	
**Specificity (95% CI)**	90% (82%–98%)	

CI: Confidence Interval.

*: Prediction of recurrence based on masked evaluation of immediate post-operative photos.

**Table 3:** Comparison of predicted versus true location of recurrence at 6 weeks post-operatively.

	Location of Recurrence at 6 Weeks					
	Nasal		Central		Temporal	
Prediction*	No	Yes	No	Yes	No	Yes
**No**	54	13	73	8	81	8
**Yes**	15	18	8	11	5	6
**Sensitivity (95% CI)**	58% (41%–75%)		58% (36%–80%)		43% (17%–69%)	
**Specificity (95% CI)**	78% (69%–88%)		90% (84%–97%)		94% (89%–99%)	

CI: Confidence Interval.

*: Prediction of recurrence location based on masked evaluation of immediate post-operative photos.

### Eyelid contour abnormalities

**Figure 4:**
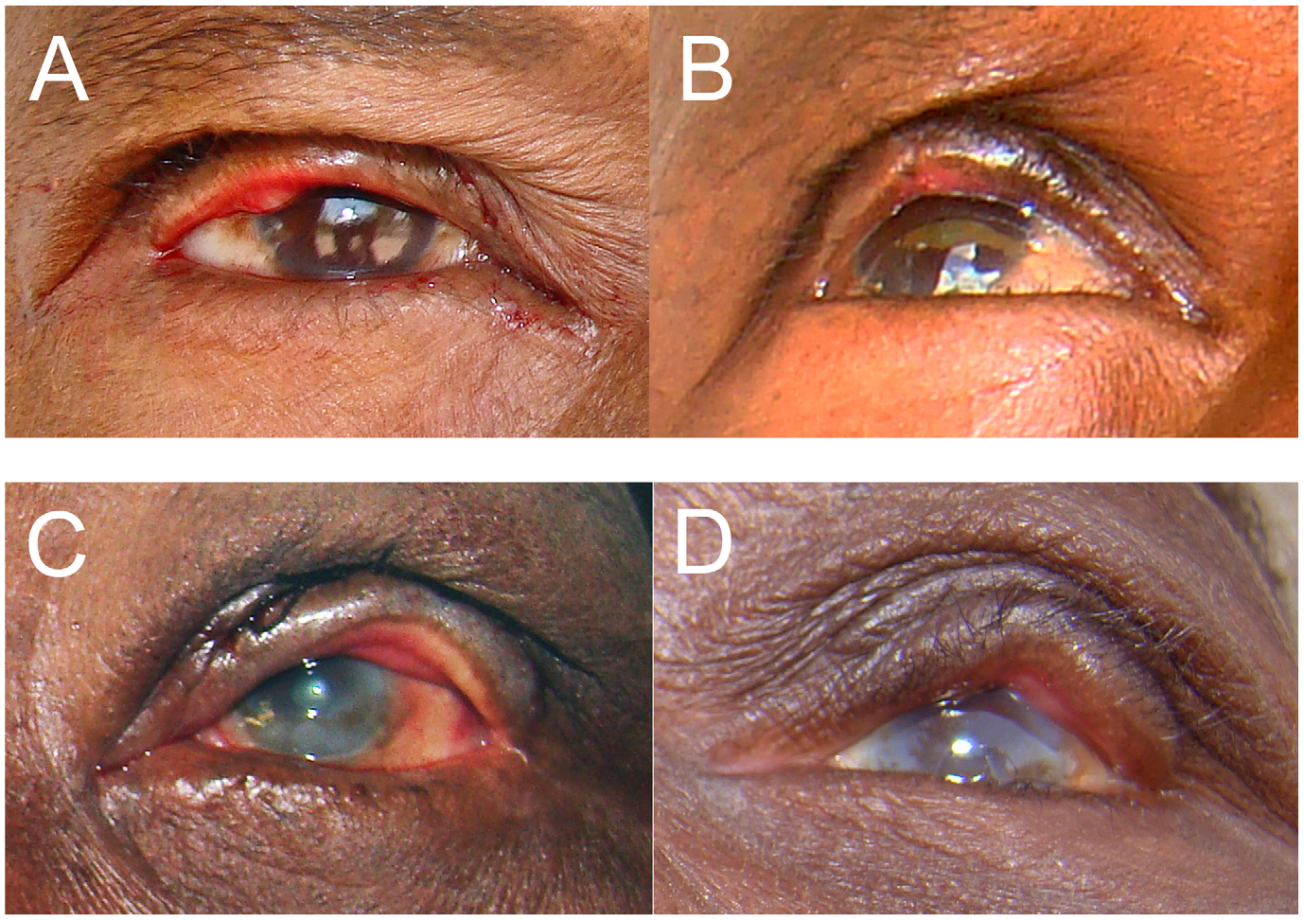
Immediate post-operative photographs used to correctly predict eyelid contour abnormalities (ECAs). A, C) Immediate post-operative photographs in 2 participants that were used to correctly predict ECAs in the corresponding 6-week photographs, (B) moderate ECA and (D) severe ECA.

The evaluator accurately identified 85 of 100 eyelids with ECAs (Figure 4, Table 4).The number of false positives and false negatives were similar. The sensitivity and specificity of predicting the presence of an ECA were 85% and 88%, respectively. In addition, the severity of the ECA at 6 weeks could also be predicted in more than half of the cases (58 of 100) based on immediate post-operative photographs (Table 5). The sensitivity of predicting either a moderate or severe ECA compared to a prediction of no ECA was 64%, and a similar number of eyes were classified as moderate when they were severe as were classified as severe when they were moderate.

**Table 4:** Comparison of predicted versus true eyelid contour abnormality (ECA) at 6 weeks post-operatively.

Prediction*	Presence of Contour Abnormality at 6 Weeks	
	No	Yes
**No**	44	15
**Yes**	6	85
**Sensitivity (95% CI)**	85% (78%–92%)	
**Specificity (95% CI)**	88% (79%–97%)	

CI: Confidence Interval.

*: Prediction of moderate or severe ECA based on masked evaluation of immediate post-operative photos.

**Table 5:** Comparison of predicted versus true eyelid contour abnormality (ECA) at 6 weeks post-operatively.

	Severity of Contour Abnormality at 6 weeks		
Prediction*	No Abnormality	Moderate Abnormality	Severe Abnormality
**No Abnormality**	44	8	7
**Moderate Abnormality**	3	28	13
**Severe Abnormality**	3	14	30

*: Prediction of ECA severity based on masked evaluation of immediate post-operative photos.

## Discussion

Although previous research has looked at various aspects leading to trichiasis recurrence, our study is the first to systematically evaluate the role of immediate post-operative eyelid contour on unfavorable outcomes. We found that the degree of correction and shape of the eyelid immediately after surgery were predictive of surgical outcome at 6 weeks, suggesting a novel strategy that could be employed to lower recurrence rates and the incidence of ECAs. It should be noted that our prediction rate was higher than the expected 25% prediction rate one would expect by chance alone.

While specificity was quite high for each outcome, sensitivity was somewhat lower. A re-review of the 15 eyelids with recurrence that was not predicted by the evaluator showed that in the majority of cases, nasal under-correction was obscured by either eyelid swelling or incomplete opening of the post-surgical eyelid. In 2 cases, the immediate post-operative eyelid position appeared ideal, but at 6 weeks the eyelid had central recurrence. These cases highlight the fact that surgical outcomes are multifaceted and the immediate post-operative eyelid contour is not the only predictor of a successful surgery. The sensitivity was lower for the exact location of recurrence than the presence of recurrence. One explanation for this finding is the somewhat subjective division of the eyelid into three segments, resulting in difficulty determining the exact location when the offending lashes are near the junction between 2 locations. By combining the central and nasal locations for analysis, the sensitivity increased to 72%. Similarly, combining central and temporal aspects also resulted in a higher sensitivity (75%).

BLTR is considered an effective surgical procedure for the treatment of cicatricial entropion of the upper eyelid [10] and is one of the WHO's procedures of choice for trachomatous entropion and trichiasis. [3] The procedure was first described by Wies for the treatment of spastic entropion of the lower eyelid. [11] A tendency towards over-correction with the Wies procedure led Ballen, in 1964, to propose the technique for difficult upper eyelid problems such as cicatricial entropion in the setting of trachoma. [2] In Ballen's manuscript, the tension of the rotating sutures or desired degree of rotation of the distal fragment was not addressed. Twelve years later, Baylis noted that over-correction with the Wies procedure generally resulted from excess rotation due to improperly placed incisions or sutures. [12] He noted that contraction of the orbicularis tended to turn the eyelid margin inward, and with time, over-correction tended to lessen, as did correction of the entropion. He suggested that, “A small overcorrection, therefore, is desirable with the Wies procedure.” [12] The WHO manual states that the sutures “should be tied firmly enough to produce a slight overcorrection,” [3] but the manual does not elaborate on exactly what represents a slight over-correction. Other manuscripts also have referred to the desired “slight over-correction” without further description, [6] and some have advocated intentional over-correction, particularly in cases of severe forms of TT. [13] However, to our knowledge, what represents a “slight over-correction” or the optimum post-operative contour has not been specifically described or evaluated in the literature. In our study, we were able to use the post-operative contour to predict the surgical outcome, predicting recurrence when apparent under-correction was observed and predicting ECA when over-rotation of the eyelid was observed. Our successful predictions suggest that a future study of how the degree of rotation affects the outcome is warranted.

**Figure 5:**
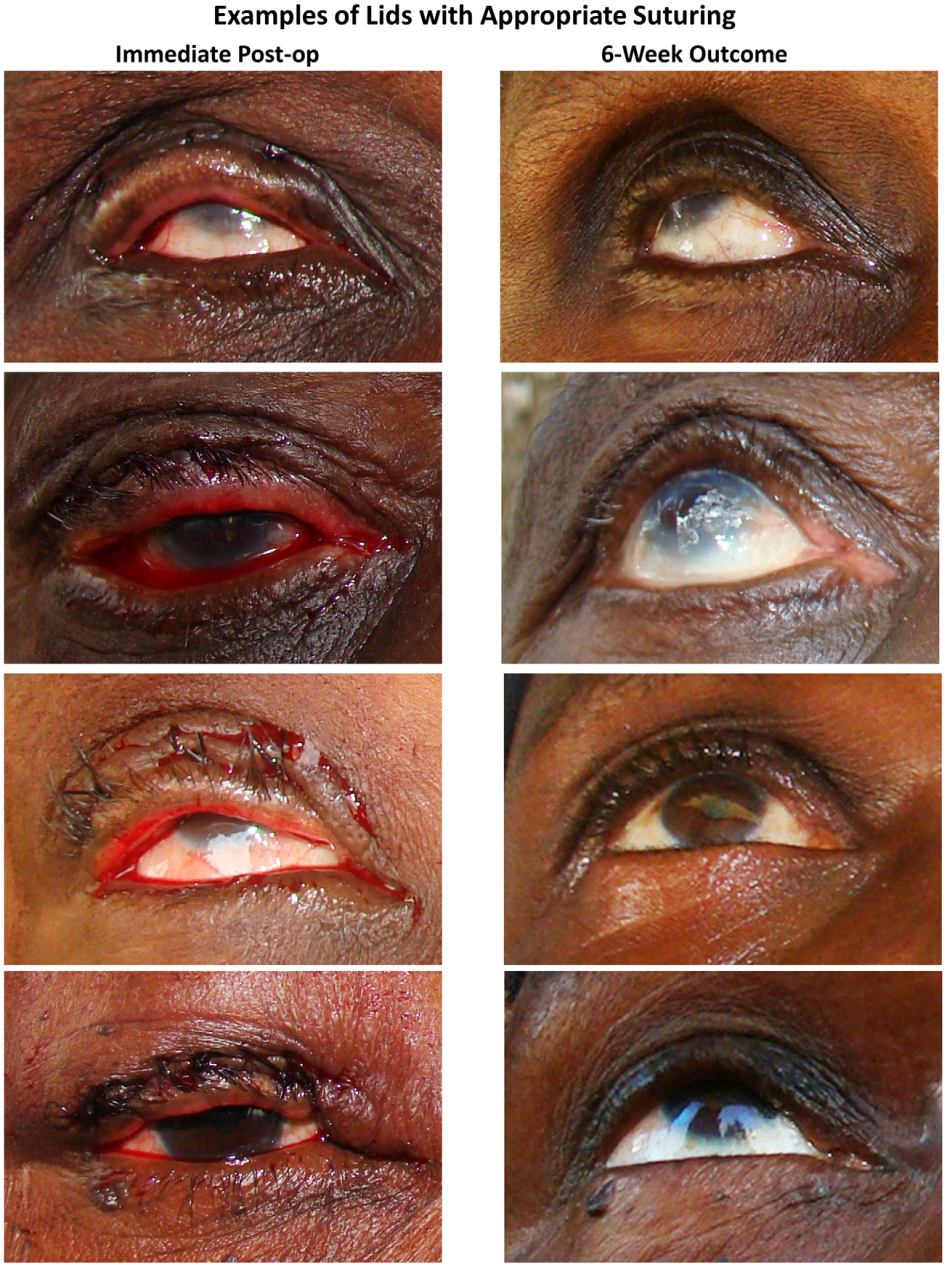
Surgical Photocard, side A, to assist surgical technicians with immediate post-operative assessment. Surgical Photocard, side A showing the immediate post-operative photographs of 4 participants without recurrence and normal contours at 6 weeks. This degree of “slight over-correction” likely represents the ideal immediate post-operative appearance to minimize both recurrence and ECAs.

**Figure 6:**
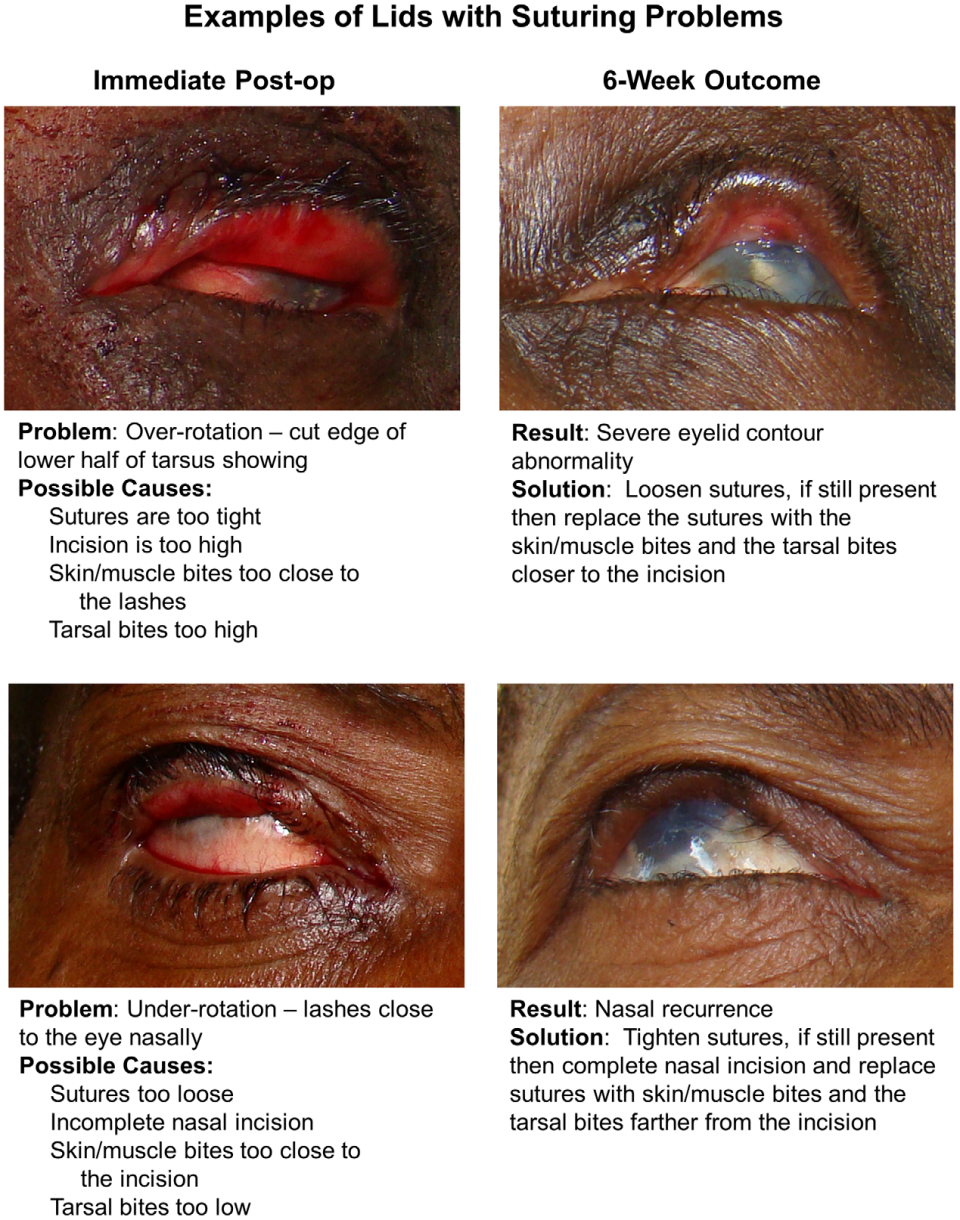
Surgical Photocard, side B, to assist surgical technicians in assessing and modifying the immediate post-operative eyelid position. Surgical Photocard, side B showing (upper left) a participant with immediate post-operative over-rotation and (upper right) the resulting severe ECA at 6 weeks and instructions as to how to improve the over-rotation; and (lower left) a participant with immediate post-operative under-rotation and (lower right) the resulting nasal recurrence at 6 weeks and instructions as to how to improve the under-rotation.

In the PRET Surgical trial, although we initially provided brief training to the surgical technicians to assess the immediate post-operative contour, we still saw unfavorable outcomes at 6 weeks. One explanation is that while an experienced oculoplastic surgeon can accurately assess the immediate post-operative outcome, non-physician surgical technicians may have more difficulty with this evaluation. However, in our experience, surgical technicians improve with additional training. Another possibility is that over time, the surgical technician reverts back to pre-training practices. Therefore, based on our data, photographs, and clinical experience, we developed a Surgical Photocard to aid the BLTR surgical technician in the field (Figures 5 and 6). We tested a prototype card during a retraining session for the PRET surgical technicians prior to commencing surgery for our participants with recurrence. This card shows examples of immediate post-operative contours that lead to successful surgical outcomes (Figure 5), as well as examples of immediate post-operative contours that lead to recurrence or ECAs (Figure 6). In the cases of sub-optimal appearance, the card also describes how to adjust suture tension or placement to improve the immediate post-operative contour based upon basic oculoplastic principles and the experience of an oculoplastic surgeon (SLM). This card can be kept with the other surgical supplies so that it is consistently brought to the field during surgical campaigns, providing a ready reference for the surgical technician.

Several barriers to improving surgical outcomes still exist. In countries where funds and supplies are limited and the need for surgery is great, convincing surgical technicians to use additional suture material and time to replace them may be difficult. Another barrier to improving surgical outcomes is the common practice of someone other than the operating surgical technician removing the sutures. When this happens, the surgical technician misses the opportunity for valuable feedback. Recent data suggests that absorbable sutures are beneficial to the ultimate outcome;[14] however, they also reduce the likelihood that the surgical technician will have an opportunity for post-operative feedback.

One limitation of this study is the use of photographs to evaluate the immediate post-operative eyelid position rather than in-person evaluation. While we have demonstrated a good agreement between field and photograph grades of postoperative outcomes at 6 weeks, [9] we have not studied the same types of correlation for the evaluation of immediate post-operative eyelids. At completion of surgery, the eyelid is still anesthetized, which sometimes makes it difficult for the participant to cooperate with the photographer and fully open or close the eyelid. Additionally, the eyelid can be quite swollen, obscuring the eyelid margin contour in a photograph. Thus, sub-optimal photographs can make it more difficult to evaluate the post-operative eyelid position than the evaluation would be in person.

Another limitation of the study is the relatively short follow-up. There is good evidence that the eyelid position continues to change and recurrence continues to occur after 6 weeks. It is possible that some of the false positive recurrences were actually recurrences at the 1- or 2-year follow-up visits and that ECAs may change over time. This study was designed to select patients with just one outcome (normal, recurrence, or ECA). While some participants had more than one adverse outcome at 6 weeks, these participants comprised only 0.5% of the gradable photographs. At the 6 week visit, only 4% of eyes had a missing or poor quality photograph.

In summary, we found that the degree of correction and shape of the eyelid immediately post-operatively were predictive of surgical outcome at 6 weeks. This finding suggests that a post-operative assessment combined with improved suturing technique could lead to better outcomes, including lower recurrence rates and a lower incidence of ECAs. Additionally, we identified immediate post-operative contours representing the “slight over-correction” that leads to a successful BLTR outcome, which if used in training and during practice has the potential to aid the TT surgical technician in their assessment.
